# Perceived Stress in Adolescence: Structure, Stability, and Its Relationship with Mental Health

**DOI:** 10.3390/ejihpe16080109

**Published:** 2026-07-23

**Authors:** Maja Ribar, Josip Razum, Goran Milas

**Affiliations:** Institute of Social Sciences “Ivo Pilar”, 10000 Zagreb, Croatia; maja.ribar@pilar.hr (M.R.); josip.razum@pilar.hr (J.R.)

**Keywords:** perceived stress, adolescence, longitudinal research design, factor structure, temporal stability, mental health, measurement invariance, well-being, depression, anxiety

## Abstract

Adolescence is a developmental period characterized by increased exposure to stress and elevated vulnerability to mental health problems. The present study examined the structure of perceived stress, its longitudinal stability, and its associations with mental health in adolescents. Data were collected from a probabilistic sample of 1679 Croatian adolescents who were in the first or second year of high school at baseline and were followed across three measurement waves approximately six months apart. Perceived stress was assessed across six domains (school, future, parents, friends, romantic partners, and self), and structural equation modeling was used to test factor structure, measurement invariance across time and sex, and temporal stability. Competing models supported a hierarchical structure comprising six domain-specific factors and a higher-order general perceived stress factor. The measure demonstrated strong longitudinal and sex invariance. General perceived stress showed high stability across the three waves, despite being assessed as a current subjective experience. Perceived stress was moderately to strongly associated with depression, generalized anxiety, internalizing and externalizing symptoms, life satisfaction, and happiness, with no evidence of sex differences in these associations. These findings suggest that adolescent perceived stress is best conceptualized as a hierarchical and highly stable construct that is closely linked to multiple indicators of mental health and well-being.

## 1. Introduction

Adolescence is a particularly sensitive developmental period, marked by a higher risk of developing mental health problems compared to childhood or adulthood (e.g., S. A. Silva et al., 2020). Therefore, understanding stress in this period can have important implications. This heightened vulnerability reflects the convergence of multiple developmental processes (Dahl, 2004). Adolescents are required to navigate major developmental tasks, including increasing autonomy from parents, establishing and maintaining peer relationships, forming a stable identity, and managing growing academic and social demands (Steinberg & Morris, 2001). At the same time, significant neurobiological changes occur, particularly in brain systems involved in emotional reactivity, reward processing, and self-regulation (Somerville & Casey, 2010). The asynchronous development of socioemotional and cognitive control systems may increase adolescents’ sensitivity to environmental challenges and stressors. Consequently, stress experienced during adolescence may have particularly important implications for both current adjustment and future developmental trajectories (Sisk & Gee, 2021).

Developmental research further suggests that experiences during adolescence can shape psychological functioning well into adulthood. Exposure to elevated levels of stress during this period has been associated with increased risk for later emotional disorders, difficulties in social functioning, and reduced well-being across the lifespan (March-Llanes et al., 2017). Understanding how adolescents perceive and experience stress is therefore important not only for explaining contemporaneous mental health outcomes but also for identifying factors that may influence longer-term developmental pathways. Therefore, understanding stress in this period can have important implications. Importantly, the same stressful event can be perceived differently, and the mere occurrence of a stressful event is not as related to mental health problems as its perceived severity (e.g., Shields et al., 2022). This study focused on subjective, or perceived stress in several domains, its structure, its stability, as well as its relationships to mental health. Subjective or perceived stress reflects individuals’ appraisal of situations as demanding, threatening, or exceeding their coping resources. According to transactional models of stress, it is this appraisal process that largely determines emotional and behavioral responses to potentially stressful circumstances (Lazarus & Folkman, 1984). Therefore, understanding perceived stress may provide a more proximal indicator of psychological functioning than exposure to stressful events alone.

While some existing instruments operationalize stress as a single domain (e.g., Depression Anxiety Stress Scales Short Form; DASS-21; Lovibond & Lovibond, 1995), The Problem Questionnaire (PQ; Seiffge-Krenke, 1995; Milas et al., 2019), used in this study, assesses multiple domains of subjective stress, and it was specifically developed for use with adolescents. Previous studies using the PQ (Seiffge-Krenke, 1995; Seiffge-Krenke et al., 2012) demonstrated that adolescent stress is multidimensional and that distinct domains of perceived stress can be identified through factor-analytic approaches. These domains correspond to major developmental contexts in adolescence, including family relationships, peer interactions, school-related experiences, and concerns about the self and future. However, evidence regarding the longitudinal psychometric properties of the measure remains limited. In particular, research examining measurement invariance across time and gender, as well as temporal stability across multiple assessments, is lacking.

In general, longitudinal psychometric investigations of adolescent stress are scarce. For example, a recent systematic review (Dwight et al., 2024) identified 18 studies that measured stress in youth populations via the DASS-21 (Lovibond & Lovibond, 1995), and only one of these studies (H. A. D. Silva et al., 2016) computed the temporal stability of stress. Moreover, that study relied on a relatively small sample and only two measurement occasions. Several other studies also reported correlations between perceived stress at different time points, which were found to be high (Byrne et al., 2006; Burger & Samuel, 2017; Dulaney et al., 2018). However, these studies did so without creating latent variable models and examining factor structure and measurement invariance.

Furthermore, when measuring stress in adolescents, it is important to study the convergent validity of a stress measure specifically in this population. Stress is expected to be associated with depression, and evidence indicates that stressful life events are causally related to the onset of major depressive episodes (Kendler et al., 1999; Harkness & Stewart, 2026), and that this relationship may be bi-directional (Harkness & Stewart, 2026). Comparatively less research has examined stress in relation to the onset of anxiety disorders (Uliaszek et al., 2010). Nevertheless, findings suggest that some anxiety disorders may be precipitated by highly threatening and uncontrollable stressors (Uliaszek et al., 2010), and that stress can prospectively predict subsequent episodes of generalized anxiety disorder (Francis et al., 2012). Several mechanisms may explain these associations. Chronic or intense stress may overwhelm coping resources, contribute to maladaptive cognitive processes such as rumination and worry, and disrupt emotional regulation (Wadsworth, 2015). Stress can also interfere with social relationships, academic functioning, and perceived competence, thereby increasing vulnerability to internalizing and externalizing problems. Consequently, perceived stress is often conceptualized as a transdiagnostic risk factor that contributes to a broad range of psychological difficulties (Hostinar et al., 2023). Consistent with this perspective, studies involving adolescents have demonstrated associations between stress and both depression and anxiety (Byrne et al., 2006; Lupo, 2023). Stressful life events in adolescents have been linked to both internalizing and externalizing symptoms cross-sectionally and longitudinally (March-Llanes et al., 2017).

When it comes to positive mental health outcomes, a study conducted in a large adult worldwide sample has found a negative relationship between stress and well-being, and the relationship was stronger for feelings than for life satisfaction (Ng & Diener, 2022). A negative relationship between stress and life satisfaction and happiness was also found in a sample of college students (Schiffrin & Nelson, 2008). Finally, life satisfaction has also been associated with lower stress in adolescents and may buffer the association between stress and maladjustment (Suldo & Huebner, 2004) or also mediate pathways through which stress relates to negative outcomes (Moksnes et al., 2016). From a theoretical perspective, elevated stress may reduce well-being by limiting opportunities for positive experiences, undermining feelings of competence and control, and increasing negative emotional states (Sigfusdottir et al., 2016).

Therefore, we expected that perceived stress in adolescents relates to depression, generalized anxiety, internalizing and externalizing symptoms and life satisfaction and happiness—at least with moderate effect sizes. Accordingly, we expected moderate associations between perceived stress and indicators of both negative and positive mental health. Previous studies also investigated the moderating effects of gender, but the evidence is mixed. For example, Shi et al. (2023) reported stronger associations between stressful life events and depression among women than men, whereas a meta-analysis in adolescents found no such moderation for stress and internalizing/externalizing symptoms (March-Llanes et al., 2017). Given these mixed findings, we aimed to explore whether stress related to mental health and whether gender is a moderator of the associations in the present study.

### Research Problem and Present Study

Despite evidence that adolescent stress is multidimensional and linked to important mental health outcomes, important gaps remain. Little is known about the longitudinal measurement properties of multidimensional stress measures in adolescence, including whether their factor structure remains stable across time and gender and the extent to which perceived stress demonstrates temporal stability. Furthermore, relatively few studies have simultaneously examined associations between multidimensional perceived stress and a broad range of indicators of both psychological distress and well-being.

Therefore, the first aim of the present study was to examine the multidimensional structure of adolescents’ perceived stress across several life domains assessed by the Problem Questionnaire and to evaluate whether this structure is invariant across gender and across three measurement occasions. In addition, we examined the temporal stability of the identified stress dimensions over time. The second aim was to investigate associations between perceived stress and depression, generalized anxiety, internalizing symptoms, externalizing symptoms, subjective happiness, and life satisfaction, as well as to explore whether gender moderates these associations.

## 2. Materials and Methods

### 2.1. Participants and Procedure

Data were collected within the larger research project Longitudinal Adolescent Stress Study (STRESS LOAD). A sample of participants from schools in Zagreb, Croatia, was used. Schools were selected using proportional random sampling based on student enrolment and stratified by school type, i.e., vocational and grammar schools. Art schools were excluded because they enroll only a small share of the student population, and many of their students also attend another secondary school. After approval from the relevant Ministry, schools were invited to participate. Fifteen schools joined the first wave, and two additional schools joined the second, resulting in a final sample of 10 vocational schools and 7 gymnasiums.

Data collection was conducted during regular school classes via a mobile application developed for this project. Before accessing the questionnaire, the participants were briefly informed about the project aims, after which they gave their informed consent by clicking the consent checkbox. For those under the age of 16, consent was also given by their parents via email. The data on perceived stress were collected in three study waves, each set approximately 6 months apart: March 2022 (T1), October 2022 (T2), and April 2023 (T3), and the data used to examine relationships with correlates were collected during the first measurement wave. At each wave, all enrolled classes of the participating schools were invited to complete the survey during regular school hours, regardless of whether individual students had taken part in previous waves. Students absent on the day of administration in a given wave could therefore rejoin the study at a subsequent wave. Responses were linked across waves by means of an automatically generated identification code. Perceived stress was measured identically at T1, T2 and T3, whereas the correlate measures were administered at T1 only. The study was approved by the ethical committee of the Ivo Pilar Institute of Social Sciences (Ref. No. 11-73/20-479).

Eligibility for inclusion in the present analyses required participation in at least two of the three measurement waves and successful completion of the embedded attention-check items. Three attention-check questions were included throughout the survey to assess response validity. These items instructed participants to select a specific response option (e.g., “Choose number 1 on this question to show that you are reading the questions”). Participants who failed one or more attention checks were excluded from the analyses because their responses raised concerns regarding data quality and attentiveness. In addition, participants who completed only one wave of data collection were excluded because longitudinal analyses required data from at least two measurement occasions.

The final analytic sample consisted of 1679 adolescents who participated in at least two of the three study waves and met the data quality requirements. Specifically, the sample included 688 boys (41.2%) and 974 girls (58.4%), while a small number of participants did not report their sex. Participants were on average 15.85 years old at T1 (*SD* = 0.66), 16.37 years old at T2 (*SD* = 0.66), and 16.80 years old at T3 (*SD* = 0.68). The sample included students from both vocational schools and gymnasiums, with vocational school students comprising approximately 60% of the sample, which closely reflects the national distribution of Croatian secondary school students. Although the sample was generally balanced with respect to sex and school type, there was a modest overrepresentation of female students and students attending gymnasiums.

### 2.2. Measures

#### 2.2.1. Perceived Stress

Perceived stress was operationalized as a subjective appraisal of problems relevant to well-being, using an adapted version of the Problem Questionnaire (Seiffge-Krenke, 1995). For the present study, we used the Croatian adaptation of the questionnaire developed by Milas et al. (2019), employing a further shortened 18-item version validated for use with Croatian adolescents (Milas et al., 2024). This abbreviated measure provides an assessment of adolescents’ current perceived stress across the six domains while reducing participant burden. The questionnaire included stressors from six domains: school, future, parents, friends, romantic partners, and self. Each domain was measured by three items, with a 5-point rating scale (1 = *not stressful at all*, 5 = *highly stressful*). An example item is: “There is a great pressure to get the best marks in school”. Students were asked to rate their current stress levels. Descriptives and reliabilities are shown in Table 1. Questionnaire items and their translation are shown in the Appendix A. Perceived stress was operationalized as the subjective appraisal of demands in specific life domains (school, future, parents, friends, romantic partners, self), whereas the mental health correlates were assessed via symptom- and evaluation-based measures (e.g., depressive and anxiety symptoms, life satisfaction). The item content of the stress measure refers to appraisals of domain-specific stressors rather than to affective or somatic symptoms, which limits content overlap at the item level.

#### 2.2.2. Depression

Depressive symptoms were measured using Croatian-language version of the Patient Health Questionnaire-9 (PHQ-9; Kroenke et al., 2001), a brief self-report instrument which scores each of the 9 DSM-IV/DSM-5 diagnostic criteria for depression that were present during last two weeks. Response scale ranges from 0 (*not at all*) to 3 (*nearly every day*). An example item is: “Feeling down, depressed, or hopeless”.

#### 2.2.3. Generalized Anxiety

Generalized anxiety was measured using Croatian-language version of the Generalized Anxiety Disorder-7 scale (GAD-7, Spitzer et al., 2006), which is a brief self-report measure of generalized anxiety symptoms, present in last two weeks. An example item is: “Feeling nervous, anxious, or on edge”. The response scale ranges from 0 (*not at all*) to 3 (*nearly every day*).

#### 2.2.4. Internalizing and Externalizing Symptoms

The Croatian-language version (Tatalović Vorkapić et al., 2017) of Strengths and Difficulties Questionnaire (Goodman et al., 1998) was used as a measure of internalizing and externalizing problems. The questionnaire consists of five scales of five items each that measure Hyperactivity/inattention, Emotional Symptoms, Conduct Problems, Peer Problems and Prosocial Behavior. The Internalizing score is the sum of the Emotional and Peer difficulties scales, and the Externalizing score is the sum of the Conduct and Hyperactivity scales. Response scale ranges from 0 (*not true*) to 3 (*certainly true*). Examples of items include: Internalizing problems (“I get a lot of headaches, stomach-aches or sickness”) and Externalizing problems (“I get very angry and often lose my temper”).

#### 2.2.5. Life Satisfaction

Life satisfaction was assessed using a single-item Croatian-language measure (Jovanović & Lazić, 2018), asking participants to rate their life satisfaction on a response scale ranging from 0 (*not satisfied at all*) to 10 (*extremely satisfied*). A large-scale study conducted across different age groups in a cultural context similar to Croatia (Jovanović & Lazić, 2018) found that a single-item measure of life satisfaction correlated highly with the multi-item scale and that its correlations with 27 other constructs were largely indistinguishable from those of the longer scale.

#### 2.2.6. Happiness

Happiness was assessed using a single-item Croatian-language measure (Lukoševičiūtė et al., 2022). Participants rated how happy they generally felt on a scale ranging from 0 (*not happy at all*) to 10 (*extremely happy*). This single-item measure is the most widely used measure of happiness in adolescent research and a large-scale study (Lukoševičiūtė et al., 2022) showed that it correlated strongly with similar constructs (e.g., well-being) and, as expected, had moderate to low negative correlations with convergent validity indicators such as health complaints, bullying, and self-harm behavior.

#### 2.2.7. Data Analysis

The structure of perceived stress was examined using structural equation modeling. Model fit was assessed using the following indices and guidelines for acceptable fit (Hu & Bentler, 1999): TLI and CLI close to 0.95, SRMR close to 0.08, and RMSEA close to 0.06. Three types of sex and longitudinal invariance were tested: configural invariance (pattern invariance), weak invariance (loading invariance), and strong invariance (intercept invariance). Although strict, or residual, invariance is sometimes investigated, this type of invariance was not tested because it is considered overly restrictive (Little, 2024). Invariance was tested in a hierarchical sequence since each more restrictive model is nested under the less restrictive model. In longitudinal models, errors of corresponding items were allowed to correlate with each other across measurement occasions (e.g., Little, 2024). When comparing nested models (used to examine sex and longitudinal invariance), the differences in CFI < 0.01 and in RMSEA < 0.015 were treated as indicators of non-difference between the models (Chen, 2007). The stability of perceived stress was examined using latent correlations derived from the established measurement model. Because stress perceptions normatively fluctuate during adolescence (Seiffge-Krenke et al., 2009), we focused on rank-order stability, which reflects the consistency of individuals’ relative standing across waves rather than the absence of mean-level change. Sex differences in perceived stress and the relationship of stress with mental health variables were examined at the manifest variable level to allow comparisons with other research results. Analyses were carried out in R statistical environment (R Core Team, 2025), using psych (Revelle, 2023) and lavaan (Rosseel, 2012) packages. Due to missing data, maximum likelihood estimation was used, and since item distributions were skewed, a robust estimator was selected (“MLR” estimator in lavaan). Robust values of model fit statistics are reported in all instances.

## 3. Results

Reliabilities and descriptive statistics for each scale are shown in Table 1. All stress subscale reliabilities were acceptable, and the scale means pointed to relatively low levels of perceived stress among participants.

### 3.1. Structure and Stability of Perceived Stress

To examine the structure of perceived stress, at each measurement wave three models were tested: (1) a single-factor model in which all items loaded on a single factor; (2) a six-correlated-factors model with each factor representing each stressor domain; and (3) a hierarchical model with six first-order factors and a single higher-order factor. Results are shown in Table 2. In each measurement wave, the single-factor model had poor fit, and both six-factor and hierarchical model fit statistics were within, or very close to, acceptable thresholds (Hu & Bentler, 1999). In both the six-factor and hierarchical model, manifest variable saturations were higher than 0.50. The six-factor model was superior to the hierarchical model according to CFI difference criteria but fit the data equally well according to RMSEA difference criteria (Table 2). The factors were highly correlated (rs across all time points ranged from 0.45 to 0.82), and the saturations of each first-order factor with the general factor across all time points ranged from 0.66 to 0.95. This, in addition to RMSEA difference, pointed to a higher order factor (e.g., Brown, 2015), and the hierarchical model was retained.

Table 3 shows results of longitudinal measurement invariance testing, which was carried out in two sets of analyses, or groups of models. In the first group of models (upper portion of Table 3), longitudinal invariance was examined at the level of the total sample, and in the second group of models (lower portion of Table 3) invariance across time and sex was examined where constraints were added simultaneously at both levels (Little, 2024). In both sets of analyses, the configural (unconstrained) model had acceptable fit, and the fit of a more restrictive model (equal loadings) was not significantly worse, indicating weak invariance. Adding the final restriction (equal loadings and intercepts) did not significantly decrease model fit compared to the less restrictive one, and strong invariance was established.

Latent correlations between the level of stress at each time point for each lower-order stressor domain and the higher-order factor of stress are shown in Table 4. Except for stress caused by a relationship with romantic partners and the one related to self, domain-level stress was quite stable. The varying and generally lower correlations of stress caused by a romantic relationship are probably a consequence of the large number of adolescents who do not have a partner at that age, which may or may not cause stress. The general stress factor showed a high degree of stability.

### 3.2. Sex Differences in Perceived Stress and Its Relationship with Mental Health

Descriptives of perceived stress (at total and domain level) separately for each sex are shown in Table 5. At each measurement wave, perceived stress in females was significantly higher than that in males.

Table 6 shows relationships of total perceived stress and its domains with mental health (measured at the same time point), as well as intercorrelations between other study variables. All relationships were in the expected direction, and effect sizes were moderate to large. Correlations between general perceived stress and the mental health constructs ranged from r = 0.31 to r = 0.60 in absolute value, well below the most stringent cutoff of 0.75 proposed for detecting discriminant validity problems (Cheung et al., 2023). Sex differences in the relationships between perceived stress and mental health indicators are shown in Appendix B. When adjusted for multiple comparisons, none of the indicators of mental health were differently related to stress in males compared to females.

## 4. Discussion

This study examined how adolescents perceive stress related to several life domains, the structure of perceived stress, as well as its stability using a longitudinal research design. It also examined how perceived stress relates to mental health criteria and whether these relationships are moderated by sex. Our results imply that perceived stress in adolescence has a hierarchical structure, with domain-related stress at a lower level, and total stress at a higher level of hierarchy. Although the perception of stress was measured at the momentary level, perceived stress was stable across three measurement waves six months apart from each other.

Other studies investigating perceived stress in adolescents with the Problem Questionnaire (Seiffge-Krenke et al., 2012) or with other measures (e.g., Østerås et al., 2018) have identified multiple interrelated stressor domains. However, they have not examined the existence of a general factor at a higher level of hierarchy, but only models with multiple domains. Results of the present study confirmed that there are multiple stressor domains but have also indicated that there is a general tendency to experience stress across all examined stressor domains.

Although a strong general stress factor accounted for the common variance across domains, the domain-specific factors remain theoretically meaningful. The existence of a higher-order stress factor does not imply that all stress domains contribute equally to adolescents’ experiences. Rather, adolescents encounter developmental challenges in several life contexts simultaneously, including school demands, relationships with parents, peer interactions, romantic relationships, and future-oriented concerns. Stress originating from these domains may differ in frequency, intensity, controllability, and developmental significance. Academic, family, and peer stressors may differ in their developmental significance because they are linked to distinct developmental tasks, including achievement, autonomy, identity formation, and social belonging (Byrne et al., 2006; Compas et al., 2017). For example, academic stress is often linked to achievement pressures and educational transitions, whereas family- and peer-related stress may be more closely connected to identity development, autonomy seeking, and social belonging. During adolescence, successful navigation of these developmental tasks is particularly important, and difficulties in any of these domains may contribute to broader perceptions of stress. The present findings suggest that these diverse stress experiences share a common underlying component, while still retaining domain-specific characteristics that may have distinct antecedents and consequences. Future research should therefore examine whether particular stress domains show differential predictive validity for specific developmental outcomes, even after accounting for the general stress factor.

High stability of perceived stress was previously demonstrated in older adults (Gomez et al., 2014), and some studies have reported high correlations between perceived stress at different time points in adolescents (e.g., Dulaney et al., 2018). Our study has confirmed these findings, while assessing multiple stressor domains, testing different plausible factor structures, and establishing measurement invariance in a latent variable solution. The existence of a general tendency to experience stress in several life domains simultaneously that is stable and invariant across sexes could indicate the dispositional nature of experiencing stress. Therefore, future studies could examine the reasons behind the stability of perceived stress in adolescents. For example, a meta-analysis by Luo et al. (2023) has shown that psychological stress perception was related to neuroticism with an average r = 0.36, making neuroticism one plausible candidate.

Furthermore, we confirmed previous findings (e.g., Lupo, 2023; Moksnes et al., 2016) and have shown on a large sample of adolescents that stress has moderate (*r* = 0.41 for externalizing symptoms) to strong (*r* = 0.67 for depression and generalized anxiety) relationships to psychopathology and well-being. We found no sex differences in how stress was related to the examined criteria. This is in line with the meta-analytic results (March-Llanes et al., 2017) for internalizing and externalizing symptoms but we also demonstrated that there are no sex differences in relationships of stress to depression, generalized anxiety, happiness and life satisfaction. This means that stress could bear an equally negative impact on both adolescent boys and girls. The strong associations between the general stress factor and indicators of mental health further support its substantive importance. This finding suggests that the higher-order stress factor captures a broad psychological liability relevant to emotional adjustment and mental health functioning, rather than merely reflecting isolated stress experiences within specific domains.

The observed associations between perceived stress and mental health indicators are also consistent with broader theoretical models linking stress to psychological functioning. Perceived stress reflects not only exposure to potentially stressful circumstances but also cognitive appraisal processes through which individuals evaluate environmental demands and their coping resources (Cohen et al., 1983; Lazarus & Folkman, 1984). Consequently, elevated perceived stress may be associated with maladaptive cognitive styles, emotion regulation difficulties, rumination, lower perceived control, and reduced resilience (Compas et al., 2017; Nolen-Hoeksema et al., 2008; Gross, 2015). These mechanisms have previously been implicated in the development and maintenance of internalizing symptoms such as anxiety and depression. At the same time, chronic perceptions of stress may deplete psychological resources necessary for maintaining positive functioning, thereby contributing to lower life satisfaction and happiness (Diener et al., 2018; Keyes, 2005). The strong relationships observed in the present study suggest that generalized stress perceptions may represent a broad psychological vulnerability factor that spans both negative mental health outcomes and positive indicators of well-being. Future longitudinal research should investigate potential mediating mechanisms, including coping strategies, emotion regulation processes, self-efficacy, personality traits, and social support, to better understand how stable stress perceptions influence adolescent adjustment over time. Despite this evidence of discriminant validity, the strong associations between perceived stress and internalizing symptoms are consistent with theoretical models positing reciprocal relations between stress appraisal and psychopathology. Moreover, some shared method and content variance cannot be fully excluded. We acknowledge this as a limitation and an avenue for future multi-method research.

The findings have several practical implications. First, the substantial stability of perceived stress suggests that elevated stress perceptions are not merely short-term reactions to situational circumstances but may characterize a subgroup of adolescents who experience persistent difficulties in managing everyday demands. School-based screening programs could therefore benefit from assessing generalized perceived stress alongside traditional indicators of emotional and behavioral difficulties. Second, because perceived stress was strongly associated with both psychopathology and well-being, interventions aimed at reducing stress perceptions may yield benefits across multiple domains of psychological functioning. Programs that strengthen coping skills, stress appraisal processes, emotion regulation capacities, problem-solving abilities, and social support networks may be particularly valuable. Finally, the absence of sex differences in the associations between stress and mental health outcomes suggests that stress-reduction and prevention programs may be broadly applicable to both adolescent boys and girls, although sex-specific risk factors for stress exposure may still warrant consideration.

This study has several strengths, namely a large probabilistic sample of adolescents, three measurement points, and use of a comprehensive measure of perceived adolescent stress. Study limitations include the fact that only self-report data were collected to assess adolescent stress. Perhaps informant data or more objective data, like biological markers, would have shown a different degree of stability through time. Although perceived stress was examined at a momentary level, measurement points were about six months apart, which was enough to show its stability, but perceived stress variability is another aspect that could be examined in future studies. Sliwinski et al. (2009), for example, suggest that not only stable traits but also the changing characteristics of the psychosocial context influence perceived stress. Therefore, future studies could explore how stable perceived stress is at shorter time lags, for example daily or weekly, as well as its time-variant predictors. A recent study (Le et al., 2026) has shown that perceived stress in young adults was moderately stable but also varied considerably from day to day, and that it was reciprocally related to experienced positive and negative affect. Finally, the data were collected among adolescents who were in the first and second years of high school at the beginning of the study, and our results call for replication in different age groups and cultures.

In conclusion, the present findings suggest that adolescent perceived stress is best conceptualized as a hierarchical construct characterized by a strong and stable general stress factor. The stability of this factor across time and sexes, together with its strong associations with mental health indicators, points to generalized stress perception as a potentially important marker of vulnerability for later psychological difficulties. Preventive and intervention efforts may therefore benefit from identifying adolescents with chronically elevated generalized stress perceptions and targeting broader stress-processing tendencies rather than isolated stress domains alone.

## 5. Conclusions

The present study provides evidence that perceived stress in adolescence is a hierarchical construct comprising both domain-specific stress perceptions and a higher-order general stress factor. The construct demonstrated strong measurement invariance across time and sex and showed substantial stability over an approximately one-year period, despite being assessed as a current subjective experience. Furthermore, perceived stress was consistently associated with multiple indicators of mental health and well-being, including depression, generalized anxiety, internalizing and externalizing symptoms, life satisfaction, and happiness.

The study contributes to the literature by demonstrating that adolescents’ stress experiences across multiple life domains can be understood as manifestations of a broader and relatively enduring tendency to perceive life circumstances as stressful. This finding is particularly important from a developmental perspective because adolescence is a period characterized by rapid biological, cognitive, social, and emotional changes, during which individuals face numerous developmental challenges and transitions. The presence of a stable general stress factor suggests that individual differences in stress perception may become established relatively early and may influence adaptation across multiple developmental contexts.

These findings also have potential lifespan implications. If generalized stress perceptions remain stable over time, they may represent an early vulnerability factor that contributes to the accumulation of mental health difficulties and reduced well-being across later developmental periods. Consequently, identifying and addressing elevated perceived stress during adolescence may help promote healthier developmental trajectories and reduce the risk of future psychological difficulties. Interventions that target broad stress-appraisal and coping processes, rather than focusing exclusively on isolated stress domains, may therefore be particularly beneficial. Future research should examine the developmental, personality, and contextual factors that contribute to the emergence, persistence, and long-term consequences of generalized stress perceptions across adolescence and into adulthood.

## Figures and Tables

**Table 1:** Reliabilities and Descriptives of Study Variables.

	α	*M*	*SD*	*Min*	*Max*	*Skew*	*Kurt*	α	*M*	*SD*	*Min*	*Max*	*Skew*	*Kurt*	α	*M*	*SD*	*Min*	*Max*	*Skew*	*Kurt*
Stressor domain																					
School	0.70	2.40	0.96	1.00	5.00	0.52	−0.43	0.72	2.50	0.95	1.00	5.00	0.23	−0.77	0.72	2.27	0.93	1.00	5.00	0.67	−0.05
Future	0.85	2.55	1.24	1.00	5.00	0.46	−0.92	0.87	2.78	1.26	1.00	5.00	0.13	−1.19	0.89	2.68	1.25	1.00	5.00	0.32	−1.03
Parents	0.79	2.12	1.07	1.00	5.00	0.88	−0.16	0.81	2.24	1.09	1.00	5.00	0.66	−0.54	0.83	2.00	1.06	1.00	5.00	1.04	0.23
Friends	0.80	2.04	1.09	1.00	5.00	0.99	0.01	0.80	2.11	1.09	1.00	5.00	0.81	−0.30	0.82	1.90	1.05	1.00	5.00	1.17	0.49
Romantic partners	0.67	1.80	0.92	1.00	5.00	1.22	0.85	0.67	1.90	0.92	1.00	5.00	1.00	0.41	0.69	1.70	0.87	1.00	5.00	1.41	1.56
Self	0.76	2.46	1.12	1.00	5.00	0.50	−0.70	0.76	2.52	1.09	1.00	5.00	0.39	−0.76	0.75	2.31	1.06	1.00	5.00	0.58	−0.49
Total stress	0.091	2.23	0.81	1.00	5.00	0.60	−0.37	0.91	2.34	0.81	1.00	4.83	0.35	−0.53	0.92	2.14	0.80	1.00	5.00	0.70	0.03
Correlate																					
Depression	0.90	8.88	6.68	0.00	27.00	0.75	−0.22														
Generalized anxiety	0.90	7.50	5.53	0.00	21.00	0.62	−0.52														
Internalizing symptoms	0.73	6.08	3.63	0.00	20.00	0.55	−0.25														
Externalizing symptoms	0.68	6.54	3.25	0.00	20.00	0.47	−0.03														
Life satisfaction	-	7.10	2.37	0.00	10.00	−0.86	0.29														
Happiness	-	6.70	2.43	0.00	10.00	−0.67	−0.09														

**Table 2:** Tested Models of Perceived Stress Within Each Time Point.

Time Point/Model	χ^2^(*df*)	RMSEA [90% CI]	SRMR	CFI	TLI
Time 1					
Single factor	1795.47 (135)	0.118 [0.113, 0.122]	0.070	0.759	0.726
Six correlated factors	432.78 (120)	0.051 [0.046, 0.056]	0.041	0.960	0.949
Hierarchical model	552.56 (129)	0.057 [0.052, 0.062]	0.049	0.945	0.935
Time 2					
Single factor	2210.18 (135)	0.128 [0.124, 0.133]	0.076	0.727	0.690
Six correlated factors	378.33 (120)	0.047 [0.042, 0.053]	0.036	0.967	0.958
Hierarchical model	486.75 (129)	0.054 [0.049, 0.059]	0.045	0.954	0.946
Time 3					
Single factor	2496.36 (135)	0.135 [0.131, 0.140]	0.075	0.719	0.681
Six correlated factors	435.65 (120)	0.052 [0.046, 0.057]	0.039	0.964	0.954
Hierarchical model	535.09 (129)	0.057 [0.052, 0.062]	0.048	0.953	0.944

**Table 3:** Longitudinal Measurement Invariance of Perceived Stress at the Level of Total Sample (Single Group), and With Equality Constraints by Measurement Point and Sex (Multigroup).

Model/Constraints	χ^2^(*df*)	RMSEA [90% CI]	SRMR	CFI	TLI	ΔRMSEA	ΔCFI
Single group: Constraints across time							
Unconstrained	2592.00 (1284)	0.030 [0.028, 0.033]	0.050	0.964	0.960		
Equal loadings	2642.54 (1318)	0.030 [0.028, 0.033]	0.052	0.964	0.960	0.000	0.000
Equal loadings and intercepts	2797.16 (1342)	0.031 [0.029, 0.033]	0.052	0.960	0.957	−0.001	−0.004
Multigroup: Constraints across time and sex							
Unconstrained	4299.41 (2581)	0.034 [0.032, 0.037]	0.068	0.949	0.943		
Equal loadings	4469.77 (2666)	0.035 [0.032, 0.037]	0.082	0.946	0.942	0.001	−0.003
Equal loadings and intercepts	4837.89 (2726)	0.037 [0.035, 0.039]	0.087	0.937	0.934	0.002	−0.009

**Table 4:** Latent Correlations Between Level of Stress at Each Time Point for Each Lower Order Stressor Domain and Higher Order Factor of Stress.

Stressor Domain	*r*_T1, T2_	*r*_T2, T3_	*r*_T1, T3_
School	0.75	0.72	0.67
Future	0.67	0.68	0.57
Parents	0.61	0.65	0.58
Friends	0.43	0.64	0.47
Romantic partners	0.24	0.32	0.37
Self	0.47	0.30	0.42
Total stress	0.78	0.78	0.69

Note. All correlations are significant at *p* < 0.001.

**Table 5:** Sex Differences in Perceived Stress.

	Males			Females					
	*n*	*M*	*SD*	*n*	*M*	*SD*	*t*(*df*)	*p*	*Cohen d*
Time 1									
School	559	2.01	1.00	756	2.95	1.25	−14.11 (1287.9)	<0.001	0.77
Future	559	1.88	0.93	755	2.30	1.13	−15.10 (1305.2)	<0.001	0.82
Parents	559	1.71	0.89	755	2.28	1.16	−7.49 (1295.8)	<0.001	0.41
Friends	559	1.62	0.79	755	1.93	0.98	−9.96 (1310.7)	<0.001	0.54
Romantic partners	559	1.97	0.92	756	2.83	1.11	6.41 (1302.1)	<0.001	0.35
Self	559	1.87	0.66	753	2.50	0.81	−15.39 (1293.6)	<0.001	0.84
Total stress	559	1.87	0.66	753	2.50	0.81	−15.53 (1297.9)	<0.001	0.84
Time 2									
School	493	2.12	0.90	699	2.76	0.89	−12.15 (1190)	<0.001	0.72
Future	493	2.19	1.09	700	3.20	1.21	−15.05 (1121.1)	<0.001	0.87
Parents	494	2.08	1.04	700	2.36	1.12	−4.44 (1107.8)	<0.001	0.26
Friends	494	1.76	0.94	700	2.36	1.12	−10.08 (1158.7)	<0.001	0.58
Romantic partners	494	1.72	0.84	700	2.02	0.95	−5.78 (1132.6)	<0.001	0.33
Self	494	2.09	0.98	700	2.82	1.07	−12.10 (1113.8)	<0.001	0.70
Total stress	559	1.87	0.66	753	2.50	0.81	−15.53 (1297.9)	<0.001	0.79
Time 3									
School	508	1.91	0.80	805	2.50	0.93	−12.15 (1195.4)	<0.001	0.67
Future	508	2.06	1.00	805	3.07	1.23	−16.14 (1233.4)	<0.001	0.87
Parents	508	1.78	0.88	805	2.15	1.13	−6.58 (1251.7)	<0.001	0.35
Friends	508	1.60	0.87	805	2.09	1.12	−9.04 (1257.5)	<0.001	0.48
Romantic partners	508	1.58	0.77	805	1.78	0.92	−4.30 (1215.4)	<0.001	0.23
Self	508	1.84	0.85	805	2.60	1.07	−14.20 (1241.2)	<0.001	0.77
Total stress	508	1.80	0.66	805	2.36	0.79	−14.03 (1214.6)	<0.001	0.76

**Table 6:** Pearson Correlations of Perceived Stress with Mental Health Variables.

Variable	1	2	3	4	5	6	7	8	9	10	11	12	13
Stressor domain													
1. School	-												
2. Future	0.50	-											
3. Parents	0.51	0.39	-										
4. Friends	0.37	0.44	0.46	-									
5. Romantic partners	0.36	0.34	0.38	0.62	-								
6. Self	0.45	0.47	0.52	0.63	0.54	-							
7. Total stress	0.71	0.72	0.74	0.79	0.71	0.81	-						
Correlate													
8. Depression	0.39	0.46	0.43	0.44	0.35	0.60	0.60	-					
9. Generalized anxiety	0.46	0.45	0.42	0.41	0.32	0.58	0.59	0.78	-				
10. Internalizing symptoms	0.38	0.35	0.39	0.46	0.31	0.51	0.54	0.66	0.64	-			
11. Externalizing symptoms	0.26	0.21	0.28	0.14	0.14	0.32	0.31	0.47	0.49	0.42	-		
12. Life satisfaction	−0.26	−0.36	−0.35	−0.35	−0.29	−0.47	−0.47	−0.55	−0.46	−0.48	−0.29	-	
13. Happiness	−0.25	−0.32	−0.34	−0.34	−0.27	−0.47	−0.45	−0.62	−0.50	−0.52	−0.34	0.76	-

Note. All correlations are significant at *p* < 0.001.

## Data Availability

The data that support the findings of this study are available from the corresponding author upon reasonable request.

## Appendix A. Original and Translated Items from the Problem Questionnaire (Seiffge-Krenke, 1995)

**Table A1:** Koliko su ti u ovom trenutku stresni sljedeći problemi [How stressful are the following problems for you at the present moment?].

Stressor Domain/Original Item	Item in Croatian
Problems related to school	
1. There is a great pressure to get the best marks in school	1. Moram imati što bolje ocjene
2. There is no comradeship in my class, only competition	2. Natjecateljski duh među učenicima, umjesto kolegijalnost
3. The teachers aren’t interested in my problems	3. Površan odnos s profesorima
Problems related to the future	
4. I don’t know what I am going to do after I finish school	4. Ne znam sto ću nakon što završim srednju školu
5. I’m unsure which profession I am best suited for	5. Nisam siguran/na koje je zanimanje najbolje za mene
6. I might become unemployed	6. Neću se moći zaposliti
Problems related to life with parents	
7. My parents show little understanding for my problems at school	7. Moji roditelji ne pokazuju razumijevanje za moje školske probleme
8. My parents are only interested in my getting good marks at school	8. Moje roditelje zanimaju samo dobre ocjene
9. I fight with my parents because my opinions about many things differ from theirs	9. Svađam se s roditeljima zbog naših različitih stavova
Problems related to relationship with peers	
10. It is difficult for me to approach others	10. Teško pristupam drugim vršnjacima
11. I don’t have a real friend with whom I can also talk about personal worries and problems	11. Nemam pravog prijatelja s kojim mogu razgovarati o osobnim problemima
12. I an unsure if the others will accept me	12. Nisam siguran/na prihvaćaju li me drugi
Problems that are related to romantic relations	
13. I feel insecure in dealing with the opposite sex	13. Nesiguran/na sam u odnosu na suprotni spol
14. I am afraid of losing contact with my other friends if I pair up with a boyfriend/girlfriend	14. Bojim se da ću izgubiti kontakt s ostalim prijateljima ako započnem vezu
15. It is difficult for me to develop a truly equal and balanced relationship	15. Teško mi je ostvariti ravnopravnu i uravnoteženu romantičnu vezu
Problems related to my own self	
16. Even little things enrage me	16. Čak me i male stvari ljute
17. I am dissatisfied with my own appearance	17. Nisam zadovoljan/na sa svojim izgledom
18. I am dissatisfied with my behavior, my own traits and abilities	18. Nezadovoljan/na sam svojim ponašanjem, osobinama i mogućnostima

Rating scale: 1 = nije stresan [not stressful at all]; 2 = malo stresan [a little stressful]; 3 = srednje stresan [moderately stressful]; 4 = vrlo stresan [very stressful]; 5 = izrazito stresan [highly stressful].

## Appendix B

**Table A2:** Sex Differences in the Relationships of Perceived Stress with Correlates.

	Males		Females		Sex Difference	
Stressor Domain/Correlate	*n*	*r*	*n*	*r*	*z*	*p*
School						
Depression	559	0.393	756	0.459	1.44	0.149
Generalized anxiety	558	0.457	756	0.505	1.12	0.264
Internalizing symptoms	558	0.376	757	0.421	0.96	0.339
Externalizing symptoms	558	0.259	755	0.279	0.39	0.700
Life satisfaction	558	−0.256	752	−0.340	1.65	0.099
Happiness	559	−0.251	754	−0.300	0.95	0.343
Future						
Depression	559	0.457	756	0.386	1.55	0.122
Generalized anxiety	558	0.445	756	0.417	0.61	0.539
Internalizing symptoms	558	0.354	757	0.325	0.59	0.558
Externalizing symptoms	558	0.214	755	0.266	0.99	0.324
Life satisfaction	558	−0.356	752	−0.334	0.45	0.656
Happiness	559	−0.317	754	−0.300	0.34	0.737
Parents						
Depression	559	0.425	756	0.493	1.54	0.123
Generalized anxiety	558	0.419	756	0.473	1.21	0.228
Internalizing symptoms	558	0.388	757	0.429	0.88	0.379
Externalizing symptoms	558	0.283	755	0.338	1.09	0.277
Life satisfaction	558	−0.347	752	−0.434	1.84	0.066
Happiness	559	−0.342	754	−0.406	1.33	0.183
Friends						
Depression	559	0.437	756	0.444	0.16	0.877
Generalized anxiety	558	0.414	756	0.392	0.47	0.639
Internalizing symptoms	558	0.455	757	0.563	2.61	0.009
Externalizing symptoms	558	0.139	755	0.180	0.75	0.452
Life satisfaction	558	−0.350	752	−0.427	1.62	0.105
Happiness	559	−0.340	754	−0.404	1.33	0.184
Romantic partners						
Depression	559	0.347	756	0.407	1.25	0.211
Generalized anxiety	558	0.318	756	0.406	1.81	0.070
Internalizing symptoms	558	0.306	757	0.408	2.09	0.036
Externalizing symptoms	558	0.136	755	0.275	2.60	0.009
Life satisfaction	558	−0.290	752	−0.325	0.69	0.490
Happiness	559	−0.267	754	−0.281	0.27	0.787
Self						
Depression	559	0.603	756	0.666	1.89	0.059
Generalized anxiety	558	0.582	756	0.625	1.21	0.226
Internalizing symptoms	558	0.512	757	0.580	1.73	0.083
Externalizing symptoms	558	0.324	755	0.451	2.68	0.007
Life satisfaction	558	−0.471	752	−0.533	1.48	0.138
Happiness	559	−0.474	754	−0.518	1.04	0.297
Total stress						
Depression	559	0.60	752	0.64	1.26	0.209
Generalized anxiety	558	0.59	752	0.63	1.14	0.252
Internalizing symptoms	558	0.54	753	0.61	1.92	0.055
Externalizing symptoms	558	0.31	751	0.40	2.00	0.045
Life satisfaction	558	−0.47	748	−0.54	1.75	0.080
Happiness	559	−0.45	751	−0.50	1.13	0.258

Note. All correlations are significant at *p* < 0.001.

## References

[B1-ejihpe-16-00109] Brown T. A. (2015). Confirmatory factor analysis for applied research.

[B2-ejihpe-16-00109] Burger K., Samuel R. (2017). The role of perceived stress and self-efficacy in young people’s life satisfaction: A longitudinal study. Journal of Youth and Adolescence.

[B3-ejihpe-16-00109] Byrne D. G., Davenport S. C., Mazanov J. (2006). Profiles of adolescent stress: The development of the adolescent stress questionnaire (ASQ). Journal of Adolescence.

[B4-ejihpe-16-00109] Chen F. F. (2007). Sensitivity of goodness of fit indexes to lack of measurement invariance. Structural Equation Modeling.

[B5-ejihpe-16-00109] Cheung G. W., Cooper-Thomas H. D., Lau R. S., Wang L. C. (2023). Reporting reliability, convergent and discriminant validity with structural equation modeling: A review and best-practice recommendations. Asia Pacific Journal of Management.

[B6-ejihpe-16-00109] Cohen S., Kamarck T., Mermelstein R. (1983). A global measure of perceived stress. Journal of Health and Social Behavior.

[B7-ejihpe-16-00109] Compas B. E., Jaser S. S., Bettis A. H., Watson K. H., Gruhn M. A., Dunbar J. P., Williams E., Thigpen J. C. (2017). Coping, emotion regulation, and psychopathology in childhood and adolescence: A meta-analysis and narrative review. Psychological Bulletin.

[B8-ejihpe-16-00109] Dahl R. E. (2004). Adolescent brain development: A period of vulnerabilities and opportunities. Keynote address. Annals of the New York Academy of Sciences.

[B9-ejihpe-16-00109] Diener E., Oishi S., Tay L. (2018). Advances in subjective well-being research. Nature Human Behaviour.

[B10-ejihpe-16-00109] Dulaney E. S., Graupmann V., Grant K. E., Adam E. K., Chen E. (2018). Taking on the stress-depression link: Meaning as a resource in adolescence. Journal of Adolescence.

[B11-ejihpe-16-00109] Dwight A. R., Briesch A. M., Hoffman J. A., Rutt C. (2024). Systematic review of the psychometric evidence supporting use of the depression anxiety stress scales, short form (DASS-21) with youth. Child & Youth Care Forum.

[B12-ejihpe-16-00109] Francis J. L., Moitra E., Dyck I., Keller M. B. (2012). The impact of stressful life events on relapse of generalized anxiety disorder. Depression and Anxiety.

[B13-ejihpe-16-00109] Gomez R., Summers M., Summers A., Wolf A., Summers J. J. (2014). Depression anxiety stress scales-21: Factor structure and test-retest invariance, and temporal stability and uniqueness of latent factors in older adults. Journal of Psychopathology and Behavioral Assessment.

[B14-ejihpe-16-00109] Goodman R., Meltzer H., Bailey V. (1998). The strengths and difficulties questionnaire: A pilot study on the validity of the self-report version. International Review of Psychiatry.

[B15-ejihpe-16-00109] Gross J. J. (2015). Emotion regulation: Current status and future prospects. Psychological Inquiry.

[B16-ejihpe-16-00109] Harkness K. L., Stewart J. G. (2026). Stress and diathesis–stress models in depression. APA handbook of depression: Classification, co-occurring conditions, and etiological processes.

[B17-ejihpe-16-00109] Hostinar C. E., Swartz J. R., Alen N. V., Guyer A. E., Hastings P. D. (2023). The role of stress phenotypes in understanding childhood adversity as a transdiagnostic risk factor for psychopathology. Journal of Psychopathology and Clinical Science.

[B18-ejihpe-16-00109] Hu L.-T., Bentler P. M. (1999). Cutoff criteria for fit indexes in covariance structure analysis: Conventional criteria versus new alternatives. Structural Equation Modeling.

[B19-ejihpe-16-00109] Jovanović V., Lazić M. (2018). Is longer always better? A comparison of the validity of single-item versus multiple-item measures of life satisfaction. Applied Research in Quality of Life.

[B20-ejihpe-16-00109] Kendler K. S., Karkowski L. M., Prescott C. A. (1999). Causal relationship between stressful life events and the onset of major depression. American Journal of Psychiatry.

[B21-ejihpe-16-00109] Keyes C. L. M. (2005). Mental illness and/or mental health? Investigating axioms of the complete state model of health. Journal of Consulting and Clinical Psychology.

[B22-ejihpe-16-00109] Kroenke K., Spitzer R. L., Williams J. B. W. (2001). The PHQ-9: Validity of a brief depression severity measure. Journal of General Internal Medicine.

[B23-ejihpe-16-00109] Lazarus R., Folkman S. (1984). Stress, appraisal, and coping.

[B24-ejihpe-16-00109] Le F., Sun T., Yap Y., Brown L. A., Wiley J. F. (2026). Temporal bidirectional dynamics of momentary stress and affect: Dynamic structural equation modelling of ecological momentary assessments. Journal of Affective Disorders.

[B25-ejihpe-16-00109] Little T. D. (2024). Longitudinal structural equation modeling.

[B26-ejihpe-16-00109] Lovibond P. F., Lovibond S. H. (1995). The structure of negative emotional states: Comparison of the depression anxiety stress scales (DASS) with the Beck depression and anxiety inventories. Behaviour Research and Therapy.

[B27-ejihpe-16-00109] Lukoševičiūtė J., Gariepy G., Mabelis J., Gaspar T., Joffė-Luinienė R., Šmigelskas K. (2022). Single-item happiness measure features adequate validity among adolescents. Frontiers in Psychology.

[B28-ejihpe-16-00109] Luo J., Zhang B., Cao M., Roberts B. W. (2023). The stressful personality: A meta-analytical review of the relation between personality and stress. Personality and Social Psychology Review.

[B29-ejihpe-16-00109] Lupo C. V. (2023). The relationship between adolescent stress in five domains and depression: Rumination as a moderator. Doctoral dissertation.

[B30-ejihpe-16-00109] March-Llanes J., Marqués-Feixa L., Mezquita L., Fañanás L., Moya-Higueras J. (2017). Stressful life events during adolescence and risk for externalizing and internalizing psychopathology: A meta-analysis. European Child & Adolescent Psychiatry.

[B31-ejihpe-16-00109] Milas G., Ćavar F., Ribar M. (2024). How much stressful life events really matter? Conceptual and methodological difficulties in assessing the impact of self-reported events on adolescents’ subjective stress. Stress and Health.

[B32-ejihpe-16-00109] Milas G., Martinović Klarić I., Malnar A., Šupe-Domić D., Slavich G. M. (2019). Socioeconomic status, social-cultural values, life stress, and health behaviors in a national sample of adolescents. Stress and Health.

[B33-ejihpe-16-00109] Moksnes U. K., Løhre A., Lillefjell M., Byrne D. G., Haugan G. (2016). The association between school stress, life satisfaction and depressive symptoms in adolescents: Life satisfaction as a potential mediator. Social Indicators Research.

[B34-ejihpe-16-00109] Ng W., Diener E. (2022). Stress’s association with subjective well-being around the globe, and buffering by affluence and prosocial behavior. The Journal of Positive Psychology.

[B35-ejihpe-16-00109] Nolen-Hoeksema S., Wisco B. E., Lyubomirsky S. (2008). Rethinking rumination. Perspectives on Psychological Science.

[B36-ejihpe-16-00109] Østerås B., Sigmundsson H., Haga M. (2018). Psychometric properties of the Perceived Stress Questionnaire (PSQ) in 15–16 years old Norwegian adolescents. Frontiers in Psychology.

[B37-ejihpe-16-00109] R Core Team (2025). R: A language and environment for statistical computing *(Version 4.6.1) [Computer software]*.

[B38-ejihpe-16-00109] Revelle W. (2023). Psych: Procedures for personality and psychological research *(Version 2.3.9) [Compute software]*.

[B39-ejihpe-16-00109] Rosseel Y. (2012). lavaan: An R package for structural equation modeling. Journal of Statistical Software.

[B40-ejihpe-16-00109] Schiffrin H. H., Nelson S. K. (2008). Stressed and happy? Investigating the relationship between happiness and perceived stress. Journal of Happiness Studies.

[B41-ejihpe-16-00109] Seiffge-Krenke I. (1995). Stress, coping, and relationships in adolescence.

[B42-ejihpe-16-00109] Seiffge-Krenke I., Aunola K., Nurmi J.-E. (2009). Changes in stress perception and coping during adolescence: The role of situational and personal factors. Child Development.

[B43-ejihpe-16-00109] Seiffge-Krenke I., Persike M., Chau C., Hendry L. B., Kloepp M., Terzini-Hollar M., Tam V., Naranjo C. R., Herrera D., Menna P., Rohail I., Veisson M., Hoareau E., Luwe M., Loncaric D., Han H., Regusch L. (2012). Differences in agency? How adolescents from 18 countries perceive and cope with their futures. International Journal of Behavioral Development.

[B44-ejihpe-16-00109] Shi J., Han X., Liao Y., Zhao H., Fan B., Zhang H., Teopiz K. M., Song W., Li L., Guo L., Lu C., McIntyre R. S. (2023). Associations of stressful life events with subthreshold depressive symptoms and major depressive disorder: The moderating role of gender. Journal of Affective Disorders.

[B45-ejihpe-16-00109] Shields G. S., Fassett-Carman A., Gray Z. J., Gonzales J. E., Snyder H. R., Slavich G. M. (2022). Why is subjective stress severity a stronger predictor of health than stressor exposure? A preregistered two-study test of two hypotheses. Stress and Health.

[B46-ejihpe-16-00109] Sigfusdottir I. D., Kristjansson A. L., Thorlindsson T., Allegrante J. P. (2016). Stress and adolescent well-being: The need for an interdisciplinary framework. Health Promotion International.

[B47-ejihpe-16-00109] Silva H. A. D., Passos M. H. P. D., Oliveira V. M. A. D., Palmeira A. C., Pitangui A. C. R., Araújo R. C. D. (2016). Short version of the Depression Anxiety Stress Scale-21: Is it valid for Brazilian adolescents?. Einstein (São Paulo).

[B48-ejihpe-16-00109] Silva S. A., Silva S. U., Ronca D. B., Gonçalves V. S. S., Dutra E. S., Carvalho K. M. B. (2020). Common mental disorders prevalence in adolescents: A systematic review and meta-analyses. PLoS ONE.

[B49-ejihpe-16-00109] Sisk L. M., Gee D. G. (2021). Stress and adolescence: Vulnerability and opportunity during a sensitive window of development. Current Opinion in Psychology.

[B50-ejihpe-16-00109] Sliwinski M. J., Almeida D. M., Smyth J., Stawski R. S. (2009). Intraindividual change and variability in daily stress processes: Findings from two measurement-burst diary studies. Psychology and Aging.

[B51-ejihpe-16-00109] Somerville L. H., Casey B. (2010). Developmental neurobiology of cognitive control and motivational systems. Current Opinion in Neurobiology.

[B52-ejihpe-16-00109] Spitzer R. L., Kroenke K., Williams J. B. W., Löwe B. (2006). A brief measure for assessing generalized anxiety disorder: The GAD-7. Archives of Internal Medicine.

[B53-ejihpe-16-00109] Steinberg L., Morris A. S. (2001). Adolescent development. Annual Review of Psychology.

[B54-ejihpe-16-00109] Suldo S., Huebner E. (2004). Does life satisfaction moderate the effects of stressful life events on psychopathological behavior during adolescence?. School Psychology Quarterly.

[B55-ejihpe-16-00109] Tatalović Vorkapić S., Slaviček M., Vlah N. (2017). Strengths and difficulties in Croatian preschool children: Validation of the strengths and difficulties questionnaire. Croatian Review of Rehabilitation Research.

[B56-ejihpe-16-00109] Uliaszek A. A., Zinbarg R. E., Mineka S., Craske M. G., Sutton J. M., Griffith J. W., Rose R., Waters A., Hammen C. (2010). The role of neuroticism and extraversion in the stress–anxiety and stress–depression relationships. Anxiety, Stress, & Coping.

[B57-ejihpe-16-00109] Wadsworth M. E. (2015). Development of maladaptive coping: A functional adaptation to chronic, uncontrollable stress. Child Development Perspectives.

